# P-1477. In vitro efficacy of Minocycline on Skin, Soft Tissue and Musculoskeletal Gram Negative bacterial isolates

**DOI:** 10.1093/ofid/ofae631.1647

**Published:** 2025-01-29

**Authors:** Patrick Crowley, Nicholas Streck, Douglas Challener, Omar M Abu Saleh

**Affiliations:** Mayo Clinic, Rochester, Minnesota; Mayo Clinic, Rochester, Minnesota; Mayo Clinic, Rochester, Minnesota; Mayo Clinic Rochester, Rochester, Minnesota

## Abstract

**Background:**

Minocycline is a second-generation tetracycline with a broad antimicrobial spectrum. It is often used in chronic suppression of musculoskeletal infections, given activity against gram positive organisms. Efficacy of Minocycline against gram negative skin and soft tissue infection has not previously been defined. In this study, we review in vitro minocycline efficacy against isolates from skin, muscle, and bone sources.

**Fig 1.**
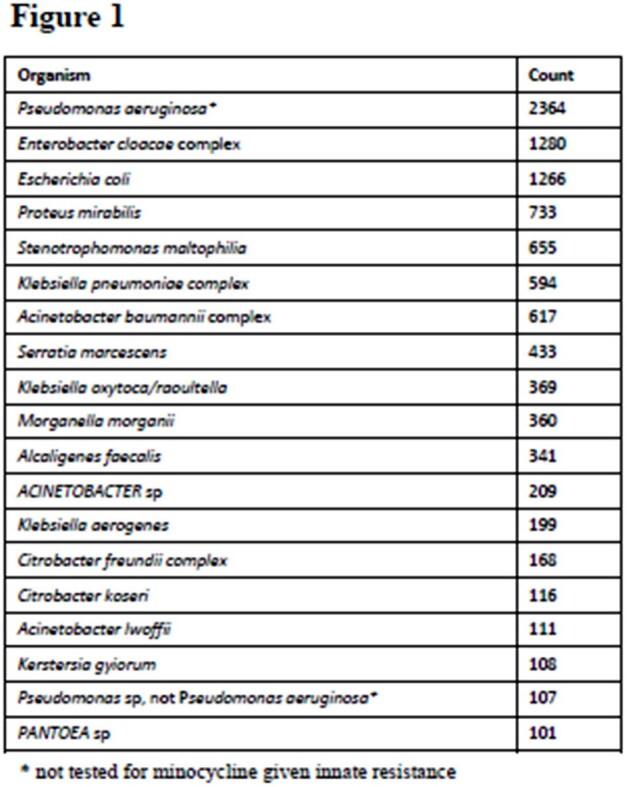
Organisms isolated

**Methods:**

We conducted a retrospective review of all minocycline susceptibility testing performed on musculoskeletal GNB isolates performed at the Mayo Clinic reference lab between 2013-2022. We describe the isolates broken down by site of culture and organism isolated. Then, we calculated the overall susceptibility of these isolates to Minocycline and susceptibility of the most common 6 organisms isolated.

**Fig 2.**
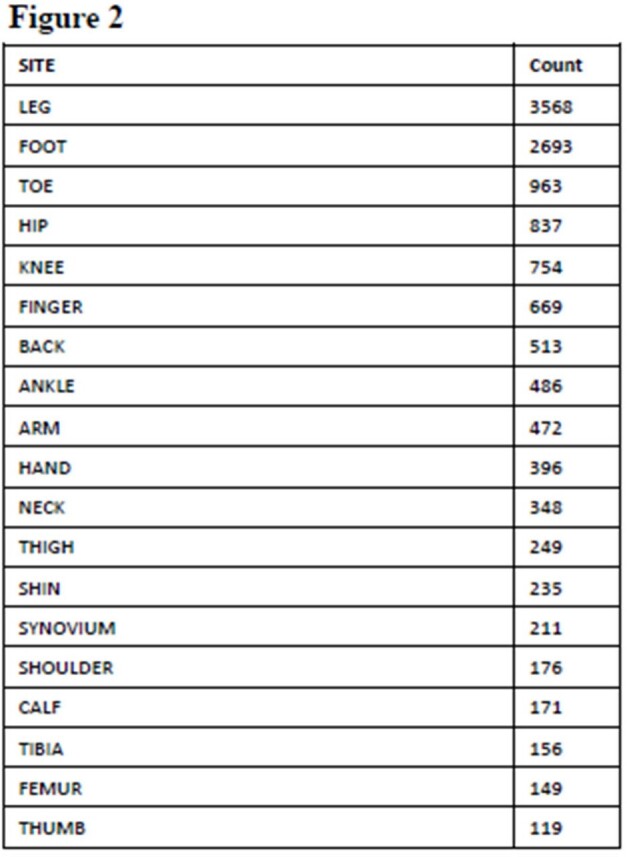
Sites of gram negative isolation

**Results:**

During the study timeframe, we identified 13,567 gram negative isolates from musculoskeletal sites. Of 330 species identified, 19 were identified in at least 100 isolates (fig 1). Of 36 unique sites, 19 sites were each identified in at least 100 isolates (fig 2). 1920 isolates were tested for susceptibility to minocycline. Of these, 1584 (82.5%) were susceptible to minocycline. Of the most common organisms identified and tested for minocycline susceptibility, 99% of Stenotrophomonas maltophilia, 83% of Enterobacter cloacae, 80% of Escherichia coli, 68% of Acinetobacter baumanii, 1% of Proteus mirabilis, 53% of Klebsiella pneumoniae, 65% of Serratia marcescens isolates were susceptible to minocycline (fig 3). Pseudomonas aeruginosa isolates were not tested, due to innate resistance.

**Fig 3.**
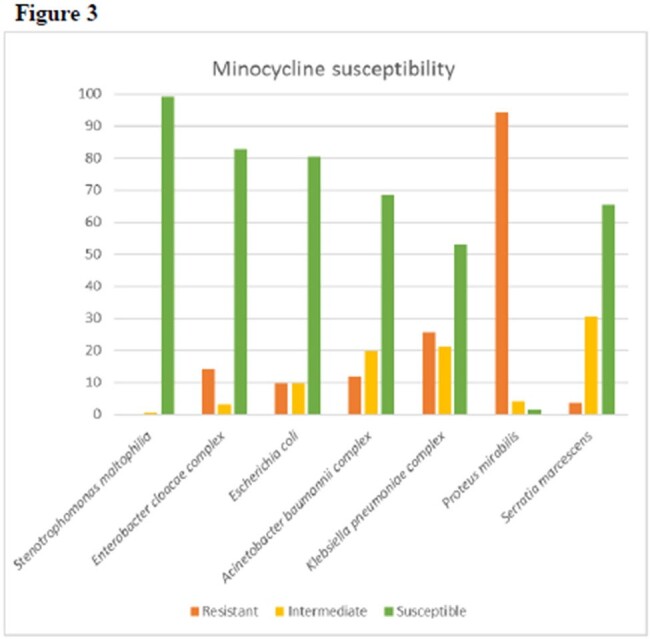
Minocycline susceptibility compared to key organisms

**Conclusion:**

A majority of skin and soft tissue cultures positive for Gram negative isolates were below the waist line. Of these, we reported organisms most commonly found. Minocycline showed in vitro activity against some commonly isolated gram negative organisms, especially E.coli, Acinetobacter, and Stenotrophomonas. Further research is needed into the efficacy of minocycline in gram negative skin and soft tissue infections, but it may represent a viable oral option, especially in the end of life.

**Disclosures:**

**All Authors**: No reported disclosures

